# Reply to Henshall et al. Commentary on “MacKechnie-Guire et al. Measuring Noseband Tightness on the Lateral Aspect of the Horse’s Face. *Animals* 2025, *15*, 537”

**DOI:** 10.3390/ani16142131

**Published:** 2026-07-09

**Authors:** Russell MacKechnie-Guire, Hilary Clayton, Jane Williams, David Marlin, Mark Fisher, Diana Fisher, Vicki Walker, Rachel Murray

**Affiliations:** 1 Equine Department, Hartpury University, Gloucester GL19 3BE, UK; jane.williams@hartpury.ac.uk (J.W.); victoria.walker@hartpury.ac.uk (V.W.); 2 Department of Large Animal Clinical Sciences, College of Veterinary Medicine, Michigan State University, 736 Wilson Road, East Lansing, MI 48824, USA; claytonh@msu.edu; 3 Animalweb Ltd., The Granary, Hermitage Court, Hermitage Lane, Maidstone, Kent ME16 9NT, UK; dm@davidmarlin.co.uk; 4 Woolcroft Equine Services, May Lane, Wisbech IP30 0DQ, UK; woolcroft2002@yahoo.co.uk (M.F.); dianafisher007@yahoo.co.uk (D.F.); 5 Ibikus Ltd., Bury St Edmunds, Suffolk IP32 7AR, UK; rmurray@ibikus.co.uk

## 1. Background

In a paper published in 2017, Doherty et al. [1] stated that “noseband tightness indices will need to be informed by ongoing evidence based equine and veterinary research relating to the impact of tight nosebands on animal welfare. Successful implementation of a tightness gauge and its acceptance by regulatory authorities and equestrian sports bodies will be very much dependent on a productive collaborative effort between equitation scientists, veterinary researchers, and the authorities and associations with interests in equine welfare”. Our study has addressed these points.

For the past several years, our research group has been engaged in studying the biomechanical interaction between the bridle and the horse’s head, particularly with reference to the adjustment of the bridle parts [2,3,4,5]. The studies have followed the suggestion of Doherty [1,6], who is an author of the letter, and have been driven by the need for evidence-based research that can be relied upon by regulatory bodies for the development of competition rules. Based on this research, the dorsal nasal planum has been selected as the optimal testing site, and the *Federation Equestre International* (FEI) has developed a tool designed to pass through the noseband in a caudal to rostral direction on the nasal planum when the noseband complies with the appropriate laxity that was informed by data [2,3,7].

The application of our research in the development of a tool designed specifically to assess noseband laxity by the FEI appears to fulfil the requirements outlined by Doherty et al. (2017) [1]. The suggestion of a need for a lateral testing site is that a small percentage of horses may resist any type of intervention on the dorsum of the nose. In the interests of the safety of the horse, handler and official, an alternative measuring site for assessing noseband compliance was considered potentially useful. Additionally, some national federations may prefer to routinely use a lateral testing site. Thus, the present study was designed as the first of a series seeking a site on the lateral aspect of the face where noseband laxity has a proportional relationship with laxity measured at the nasal planum. The next manuscript in the series will address problems with using lateral facial locations that became apparent in this preliminary work.

This letter corrects the mistakes and misinterpretations made by Henshall et al. [8] in their letter criticising our recently published manuscript entitled *Measuring Noseband Tightness on the Lateral Aspect of the Horse’s Face*. The purpose of our reply is to ensure the accurate representation of the data and methodology and to contribute constructively to the evidence base on noseband use from an objective, factual, and scientifically rigorous perspective.

## 2. Noseband Fit

Henshall et al. [8] preface their critique by emphasising the effects of excessively tight nosebands on equine welfare [9,10]. Henshall et al. [8] cite previous studies to suggest that tight nosebands may contribute to the development of oral lesions [10] and long-term pathological changes to anatomical structures beneath where the noseband is positioned [11]. While it is accepted that tight nosebands are unacceptable, the cited studies do not explicitly establish a causal relationship between noseband tightness, oral lesions [10], or anatomical changes [11], and in contrast the removal of the noseband was associated with an increased risk of oral lesions [10]. Bridle–horse interaction is complex and multifactorial, and more data is needed to quantify the nuances of the interaction. Throughout the commentary, Henshall et al. [8] omit to discuss the effects of a correctly fitted noseband, adjusted to a level that does not cause harm and that is endorsed and regulated in equestrian sports. When appropriately adjusted, a noseband serves a functional role, and the consideration of its correct use is therefore warranted to provide a balanced, non-biased perspective on noseband use.

## 3. Causing Pain and Discomfort

Henshall et al.’s [8] commentary states that “research continues into noseband use that involves repeatedly exposing horses to tightness levels that severely restrict their ability to perform these behaviours and which may cause pain and discomfort”, referring to our study. Henshall et al. [8] suggest that our studies caused pain and discomfort, a suggestion we categorically refute. Our study [7] received institutional ethical approval and confirmation from the United Kingdom Home Office that the research did not require regulation under the Animal (Scientific Procedures) Act 1986. This aligns with the fact that, at a 0.0 finger equivalent (Fe) tightness (our tightest setting studied), the noseband rests against the skin without compressing the soft tissues. A tight noseband—adjusted to the point where the soft tissues are compressed, mechanically restricting oral movement—is a noseband setting that is not recommended in training horses nor is quantitative data needed to confirm that a tightness of that magnitude is unacceptable.

We measured the distance between the inner surface of the noseband and the lateral aspect of the horse’s head when the noseband was adjusted from 2.0 to 0.0 Fe. As demonstrated in Clayton et al. [2], who used the same noseband adjustment protocol, at 0.0 Fe, horses were able to separate their jaws, accept a large treat, and chew [2]. The horses’ chewing cycle frequency remained consistent across all noseband adjustments [2]. Neither the blink rate nor eye temperature changed across conditions, suggesting that noseband adjustments did not cause a physiological response when standing or chewing [2]. While we are not advocating that 0.0 Fe is an acceptable tightness, we challenge the suggestion that the nosebands were tight enough to cause pain or discomfort. To limit misinformation, it is important that Hensall et al., [8] and others [12,13], distinguish the difference between a noseband fitted correctly vs. a noseband that is intended to restrict oral activity, as the two are vastly different.

## 4. Measuring Device Dimensions

**Figure 1:**
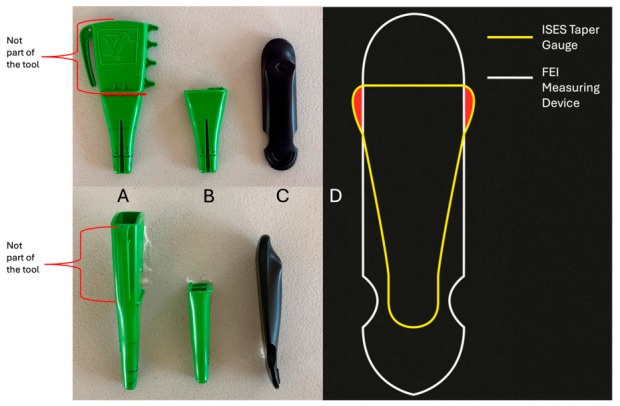
The ISES taper gauge (**A**), the part of the ISES taper gauge that is admitted beneath the noseband (**B**) and the FEI measuring device (**C**) in a plan view (**upper left**) and side view (**lower left**), with the red line indicating the portion of the tool that does not pass beneath the noseband. (**B**) (**lower**) illustrates the height of the ISES taper gauge (1.6 cm) that is admitted beneath the noseband compared with the FEI measuring device (**C**) (1.7 cm), and (**D**) illustrates an overview of the ISES Taper Gauge for the part of the tool that is inserted beneath the noseband (yellow) and the FEI Measuring Device (white) that passes beneath the noseband. The shaded red area indicates the difference in width between the two measuring devices.

Henshall et al. [8] and colleagues misinterpret the effect of the dimensions and geometry of the FEI measuring device on the clearance between the face and noseband. Previous work from our group demonstrated that tightening the noseband from 2.0 to 1.5 Fe caused no significant differences in dorsal nasal or mandible pressures in high-level dressage horses when trotting [3]. During the development of the measurement device, the FEI scrutinised published scientific research describing sub-noseband pressures to determine an appropriate degree of laxity. In a cadaver-based study, noseband pressures rose rapidly when the noseband was tightened beyond 1.4 Fe [14], providing further support for the FEI tool dimensions, which are greater than 1.5 Fe. Henshall calculated a *“24% reduction in the space under the noseband”* with the FEI measuring device [compared to the ISES taper gauge) (Figure 1).

**Figure 2:**
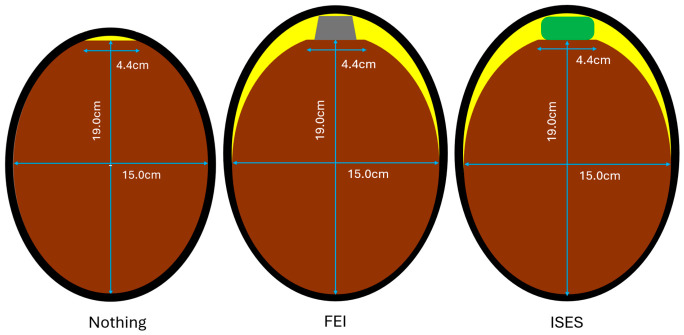
Representations of the cross-section of the head at the level of the noseband. The brown area represents the horse’s head, the black line (0.6 cm) the noseband, and the grey and green shapes the FEI measuring device and ISES taper gauge in the cross-section at the level of the maximum cross-sectional area, all to scale. The scale is indicated in cm. The yellow area represents the space between the head and the noseband (minus the gauge CSA).

In the real-life situation where the gauges do not extend beyond the essentially flat area of the nasal plane, the primary determinant of how high the noseband is lifted off the face, and therefore the noseband tightness, is the height and shape of the gauges, not simply the cross-sectional area (CSA). Using geometrical and trigonometrical analysis, in the “nothing” scenario (Figure 2), the noseband circumference is 53.6 cm (Figure 2). The FEI measuring device adds 3.4 cm to 57.0 cm, and the ISES gauge adds 3.2 cm to 56.8 cm. The FEI gauge (even though it has a slightly less yellow area) forces the noseband further away from the nasal bone overall, increasing the elliptical circumference by 2 mm compared to the ISES gauge because it is 0.1 cm taller. The ISES gauge creates more local yellow gap areas (~6.0 cm^2^ vs 4.7 cm^2^), but the overall circumference increase is marginally smaller due to its lower height. For all practical purposes, the difference in the space between the noseband and the horse’s head will not differ between FEI and ISES gauges and is likely influenced by other factors. Furthermore, in 236 horses that passed the noseband check during a tack and equipment assessment (using the FEI measuring device), mouth opening was reported as the most prevalent behaviour during competition. This suggests that the laxity of the noseband [complying to the dimensions of the FEI measuring device] did not restrict the horse’s ability to display oral activity [15]. Henshall et al. [8] question the rationale for assessing half-finger increments; this has been justified elsewhere [13]. However, other studies conducted by the authors of [8] have previously investigated noseband tightness using half-finger increments [6,16], with a presumably similar reasoning to us and the goal of understanding the nuances between noseband adjustments.

## 5. Informing Regulation

The new FEI noseband measuring device is designed to be used over the dorsal nasal bone as the standard measurement location. Prior to May 2025, however, the FEI guidelines stated that *“It must be possible to place at least one finger between the horse’s cheek and the noseband. Fingers are to be placed side by side, flat against the horse’s cheek”* (FEI Rules and Regulations, accessed 2024). Indeed, some national sport governing bodies still evaluate noseband laxity at a lateral site. Henshall et al. [8] suggest our study, through its design, methodology, and interpretation, could *“expose horses to the welfare risks associated with overly tight nosebands”* by using a lateral measurement location. The FEI’s previous methodology for determining noseband tightness appears to have been misunderstood or overlooked by these authors [8].

## 6. Noseband Selection

In our study, bridles and nosebands were fitted by two qualified bridle fitters. Henshall et al. [8] comment on the selection of nosebands used in our study. The most frequently used nosebands are the cavesson [17] and flash nosebands [6,10] in a mixed riding population (dressage, jumping, eventing and recreational). Welfare should apply to all equestrian sports; unfortunately, disproportionate attention is often given to noseband use in dressage, where the Swedish noseband is widely used [16] and referred to as “kinder” [17] due to the padding. There is little difference in the pressure distribution beneath different types of nosebands [3] when adjusted from 2.0 to 0.5 Fe. Henshall et al. [8] suggest that the findings from our study cannot be applied to other types of nosebands. We disagree, as the cavesson portion of the noseband is similar across the flash, cavesson, and Swedish designs. A flash noseband was included in pilot testing, where it was found that the distance between the inner surface of the flash noseband and the lateral aspect of the horse’s head was comparable with a cavesson noseband of the same type and tightness.

In our study [7], we acknowledged that the drop noseband was not amenable to lateral measurements, which is useful information, as prior to May 2025, noseband checks were conducted on the lateral aspect of the horse’s head regardless of the noseband type. Henshall et al. [8] suggest that our study does not apply to horses wearing a double bridle; we disagree with this assumption, as the noseband encircles the maxilla and mandible regardless of the bridle type.

## 7. Soft Tissues of the Maxilla and Mandible

Our motivation for assessing the distance between the noseband and the lateral aspect of the horse head [as was standard practice prior to May 2025] was that this approach could underestimate the tightness of the noseband over the dorsal nasal bone [7]. Henshall et al. [8] state that *“The currently accepted noseband checking site, the nasal bone, is clearly a site with minimal soft tissue between the skin and underlying bone, making its use for this purpose repeatable.”* Our data were needed to evaluate and compare possible lateral sites prior to the selection of the dorsal nasal site.

## 8. Behavioural Analysis

Henshall et al. [8] comment on the lack of behavioural analysis in our study, but they appear to have overlooked our acknowledgement of this limitation in the study: *“This study did not include a comprehensive behavioural assessment. It would be useful to study the horse’s behaviour and facial expressions while making measurements of different nosebands”.* While the study of welfare parameters is valuable [18,19], the nature of the current study required researchers to touch the horse’s head and bridle, which is likely, per se, to affect facial expressions and behaviour.

## 9. Conflicts of Interest

Henshall et al. [8] state that we did not adequately disclose the FEI’s involvement in our study. In accordance with the journal’s guidance on disclosing conflicts of interest, we explicitly stated that this body of work was funded by the FEI. For clarity, the FEI had no involvement in the study design, data collection, or interpretation of the results. Their motive was to have experts gather and evaluate data that could be used to develop a tool for measuring noseband tightness in the field.

Applying the same critique as Henshall et al. [8], it is noteworthy that potential conflicts of interest were not declared in their commentary [8]. Five of the eight authors are affiliated with the International Society for Equitation Science (ISES) (https://www.equitationscience.com/meet-the-team, accessed 1 February 2026), an organisation that develops and sells the ISES taper gauge, which is extensively referenced and endorsed in their critique. McGreevy and colleagues [20] informed the design of the ISES noseband taper gauge, which is sold and distributed by the ISES (https://www.equitationscience.com/store, accessed 1 February 2026). Given the relevance of the ISES taper gauge to the area under discussion, and its commercial and professional implications, we believe these connections should have been transparently disclosed. This would allow readers to make a fully informed assessment of the motivations and potential biases underlying Henshall et al.’s [8] commentary.

## 10. Conclusions—A Significant Step Forward

We hope that this response addresses some of the misinterpretations and oversights made by Henshall et al., many of which have provided us with an opportunity to contribute meaningfully to the broader discourse on noseband use. Again, we must address, in the strongest terms, the suggestion by Henshall et al. [8] that our study compromised equine welfare, a claim we categorically refute. Notably, the *Fédération Equestre Internationale* (FEI) has implemented a significant welfare-driven measure of restricting noseband tightness to between 2.0 and 1.5 Fe, a significant positive step for improved horse welfare. Additionally, the measurement site has been standardised to the nasal bone, rather than the lateral aspect of the head guidance based on studies that include the one critiqued here by Henshall et al. A clear pass/fail protocol has been established (1 May 2025, https://i.nside.fei.org/system/files/FEI%20Measuring%20Device-General%20Protocol%20with%20Discipline%20Protocols-Clean-17Feb2025-With%20Discipline%20logos.pdf. Accessed 1 June 2025). While critical dialogue is essential for advancing scientific understanding, it is equally important that such critique does not hinder further progress.
